# Insulin injection practice and health related quality of life among individuals with diabetes at Tikur Anbessa Specialized Hospital, Ethiopia: a cross-sectional study

**DOI:** 10.1186/s12955-023-02123-z

**Published:** 2023-05-04

**Authors:** Zenebe Negash, Aklasiya Tadiwos, Eliyas Mulatu Urgessa, Gebremedhin Beedemariam Gebretekle, Ephrem Abebe, Atalay Mulu Fentie

**Affiliations:** 1 College of Health Sciences, School of Pharmacy, Department of Pharmacology and Clinical Pharmacy, Addis Ababa University, Addis Ababa, Ethiopia; 2 College of Health Sciences, School of Medicine, Department of Dermato-Venereology, Addis Ababa University, Addis Ababa, Ethiopia; 3 Institute of Health Policy Management and Evaluation, University of Toronto, Toronto, Canada; 4 University Health Network, Toronto Health Economics and Technology Assessment (THETA) Collaborative, Toronto, Canada; 5 College of Pharmacy, Purdue University, West Lafayette, IN USA; 6 School of Medicine, Indiana University, Indianapolis, IN USA

**Keywords:** Insulin therapy, Diabetes mellitus, Quality of life, Insulin injection practice, Ethiopia

## Abstract

**Background:**

Insulin therapy can be inconvenient, painful, burdensome, and restrict patients' daily activities and health related quality of life (HRQOL) due to improper injection techniques or the nature of administration.

**Objective:**

This study aimed to assess insulin injection practice, HRQOL and predictors among patients treated with insulin at Tikur Anbessa Specialized Hospital (TASH).

**Methods:**

An institutional-based cross-sectional study was conducted among diabetes patients on insulin therapy from May to June 2022. A structured questionnaire was used to collect patient characteristics and insulin injection practice. The validated Amharic version of an EQ-5D-5L tool was used to assess the HRQOL. The data was analyzed using SPSS version 26. The patient data were summarized using descriptive statistics. One-way ANOVA using Kruskal–Wallis H tests was used to assess factors that predict insulin handling practice scores. Multivariate linear regression analysis was used to assess factors affecting HRQOL among diabetes patients treated with insulin. The EQ5D-5L utility scores of the patients were calculated using disutility coefficients taken from the Ethiopian general population. Statistical significance was declared at *p*-value < 0.05.

**Results:**

Of 319 patients who agreed and completed the survey, 51.1% of them were males. Almost half of the participants (*n* = 158) were > 50 years of age. Among the study participants, 62.1% were only on intermediate acting insulin. A significantly higher proportion of participants 291(91.2%) in this study were taking insulin two times per day. Most of the participants 234(73.4%) had fair practice with a median insulin handling practice score of 38 out of 56. Patient characteristics such as age, educational status, occupation, disease duration, and type of diabetes were significantly association with insulin injection practice (*p* < 0.05). The mean ± SD utility score of patients were 0.89 ± 0.19 (ranged from -0.04 to 1). Being female (β = -5.42, 95%CI:-8.63,-2.21, *p* = 0.001) and treated for type-I diabetes mellitus (β =  + 9.04, 95%CI: 4.23,13.85, *p*-value < 0.0001) were significantly associated with HRQOL of patients on insulin therapy.

**Conclusion:**

The study participants had fair practices in insulin handling, storage, and administration techniques, and it was seen that male and type one diabetes patients have a better quality of life compared to their counterparts.

## Introduction

The burden of diabetes mellitus (DM) has become a major public health problem and is steadily increasing in developing as well as developed nations [1, 2]. According to the 9th edition of the International Diabetes Federation (IDF) report, the global prevalence of DM in 2019 was estimated at 9.3% (463 million cases) and is expected to rise to 10.9% (700 million cases) by 2045, with 75% of patients with DM living in low and middle-income countries [3]. About 54% of people living with DM don’t know that they have diabetes in Africa, compared with 25% in North America and Caribbean countries. Moreover, Ethiopia is one of the top five countries by the number of DM patients(1.9 million people) in sub-Saharan Africa next to South Africa (4.2 million), Nigeria (3.6 million), and Tanzania (2.9 million) [4].

Despite the fact that the prevalence of DM is rising, insulin replacement therapy remains the cornerstone of care for people with type I-DM and uncontrolled type II-DM [5]. Different studies reported that poor injection technique, handling, and storage practices were common, and are the important and modifiable reasons for inadequate glycemic control [6–9]. Faulty injection technique is associated with injection-site complications, including lipohypertrophy (LH). Moreover, improper handling of needles and other sharps used in insulin injection may increase the risk of accidental injury and transmission of blood-borne infections in patients and their close contacts [10]. Until recently, many of the recommendations on insulin administration worldwide had little or no scientific underpinning and were based as much on habit and tradition as on evidence [11]. The recommended site of insulin injection subcutaneously are upper arm; and the anterior and lateral aspects of the thigh, buttocks, and abdomen at a 90° angle. However, for thin individuals or children, it is advised to use short needles and inject at 90° subcutaneously on the above mentioned sites or may need to pinch the skin and inject at a 45° to avoid intramuscular injection, especially in the thigh area. Intramuscular injection is not recommended for routine injections and rotation of the injection site is important to prevent LH or lipoatrophy [12].

In another study conducted in Ethiopia showed that the overall knowledge of the study patients regarding insulin self-administration was suboptimal and gender, marital status, occupation, area of residence and educational status were associated factors of patients’ knowledge [13]. Another study reported from northwest Ethiopia showed that even though, the study results indicated that the patients’ knowledge and practice level were moderately adequate and fair, respectively, their practical skills were significantly poor. The patients were unwilling to practice what they had already known and counseled by professionals, or they had forgotten and faces difficulties in remembering all critical steps [14]. The study in Bangladesh showed that dependence on others (family members and paramedics) for injections was also a barrier to multiple daily insulin injections [12]. The psychological barriers to initiating or intensifying insulin therapy are well known and include a fear of reduced quality of life. Although patients are generally more receptive to changing insulin regimen than to initiating insulin, some psychological barriers still exist, including perceived effects on daily activities, the burden of an increased number of injections and worry about weight gain [11].

Health-related quality of life (HRQOL) has been also associated with a number of characteristics, including poorer income, hypoglycemic episodes, low satisfaction with social support, uncontrolled blood glucose levels, age, gender, body weight, and acute and chronic diabetes complications [15]. Moreover, insulin therapy by itself can have both positive and negative impacts on HRQOL. Insulin therapy generally provides better glycemic control and improves HRQOL by reducing diabetic complications. However, insulin therapy can be inconvenient, painful, and burdensome, and can restrict patients' daily activities. Furthermore, insulin treatment sometimes induces hypoglycemic episodes, which detract from HRQOL, both in terms of the actual events and the fear they cause [16]. Therefore, improving patients’ perceptions and acceptance of insulin should be a primary goal of diabetes care [11]. Even though insulin injection practice had an impact on glycemic control and HRQOL, it was not well explored among adult patients treated at TASH, Ethiopia. Hence, the purpose of this study was to explore the insulin injection practice, and HRQOL of adult diabetes patients who are on insulin at the diabetes clinic of TASH. The study also assesses factors associated with HRQOL among diabetes patients on insulin therapy. The finding will assist researchers, policymakers, and other concerned bodies to design and develop the strategies to improve medication therapy to improve the desired treatment outcomes such as good glycemic control and improve HRQOL.

## Methods

### Study design and population

An institutional-based cross-sectional study design was applied to assess insulin injection practice and HRQOL among adult diabetes patients (both type I and II) who were on insulin from May to June 2022 at the DM clinic of TASH, Addis Ababa, Ethiopia. The clinic provides diabetes care for ambulatory patients twice a week. In addition, the clinic also provides diabetes foot care once a week for patients with diabetes foot ulcers. On average, about 6000 patients attend the diabetes clinic annually, with an average of 250 diabetes patients per week. All diabetes patients that were on follow-up at TASH and on insulin were the source population, while those who fulfilled the inclusion criteria during the study period were the study population.

### Eligibility criteria

All adult diabetes patients actively taking insulin, attending the clinic for follow-up during the study period and willing to participate in the study were included in the study. Diabetic patients who are only on oral hypoglycemic agents and gestational DM patients were excluded.

### Sample size and sampling technique

Sample size was determined based on a single population proportion formula assuming a prevalence (p) of overall good insulin injection practice was 64.3% as per a similar study conducted in the northern part of Ethiopia [14]. A z-value of 1.96 was used at 95% CI and margin of error of 5%. (*n* = sample size, *p* = prevalence, d = margin of error).

$$n\hspace{0.17em}=\hspace{0.17em}\frac{Z{\frac\alpha2} 2*p* (1 - p)}{d^{2}}= \frac{1.962*0.643(1 -0.643)}{0.052} \hspace{0.17em}=\hspace{0.17em}353$$

Due to difficulties in drawing a sampling frame related to the nature of the clinic and poor documentation who takes which medication before the patient sees the doctor, non-probability sampling (consecutive or convenient sampling) technique was used to recruit the study participants.

### Data collection and management

Data were collected using a standardized questionnaire that was prepared after reviewing various types of literature. The data abstraction and insulin injection practice assessment tools adapted from similar studies conducted in Ethiopia [14] and other published literature on similar topics [17–19]. The data collection tool was enriched by external experts’ comment. The EQ-5D HRQOL assessment tool used in this study was already validated among Ethiopians using Amharic language (Ethiopian national language) [20, 21]. The questionnaire has three sections. Section I contains socio-demographics (age, sex, place of residence, occupation, and education status), clinical (type of DM, duration since diagnosis was made, presence of other comorbidities, and blood sugar control status), and treatment-related (duration since insulin treatment started, frequency and dose of insulin, and type of insulin used) characteristics of study participants. Section II is for assessing insulin injection practice and Section III is for evaluating HRQOL using 5-level EuroQol 5 dimensions (EQ-5D-5L).

The insulin injection practice questionnaire contained 14 items, which is a Likert scale type (Never = 1; Sometimes = 2; Often/Usually = 3; Always = 4). Based on this, the injection was classified as poor practice: < 50% (scored < 28 out of 56 points); fair practice: 51–75% (scored 29–42 out of 56 points); and good practice: > 75% (scored > 42 out of 56 points) [14]. The EQ-5D-5L questionnaire consists of two parts: a descriptive system and a visual analog scale (EQ VAS). The descriptive part comprises five dimensions: mobility (MO), self-care (SC), usual activities (UA), pain/discomfort (PD), and anxiety/depression (AD). Each of the EQ-5D-5L items has five possible levels, of which four are common to all dimensions: (1) no problems, (2) slight problems, (3) moderate problems, and (4) serious problems. The fifth answer for the dimensions MO, SC, and UA was formulated as incapacity, and for PD and AD as an extreme feeling. The EQ VAS records the patient’s self-rated health on a vertical visual analogue scale, where the endpoints are labeled ‘*The best health you can imagine*’(EQ VAS score 100) and ‘*The worst health you can imagine*’ (EQ VAS score 0).

Participants were individually asked by the data collector in Amharic. The data collection tool was initially developed by English language, translated into Amharic by a fluent bilingual linguistic expert, and then back translated to English independently by the research team to check consistency. For the HRQOL, the validated Amharic Version of EQ-5D-5L was used [21]. To assure the quality of data, pre-tested on five percent of the sample were used and based on the results from the pre-test, amendments were made to the questionnaire. The training was also provided for data collectors.

### Data analysis

Collected data were checked for completeness, cleaned, entered, and analyzed using SPSS version 26. The frequency and percentages of the data were summarized using descriptive statistics. One-way ANOVA using Kruskal–Wallis H tests was used to assess the relationship between insulin handling practice scores and predictor variables. Following bivariate analysis, multivariate linear regression analysis was used to assess factors affecting HRQOL among diabetes patients treated with insulin. Patients’ EQ5D-5L utility scores were computed using disutility coefficients obtained from the Ethiopian general population [20]. *P*-value < 0.05 was used to declare a statistically significant association between dependent and independent (predictor) variables.

## Results

### Respondent characteristics

Out of 319 patients who agreed and completed the survey making a response rate of 90.4%, the number of male participants were 163(51.1%). Nearly half of the participants (*n* = 158)were aged above 50 years old. All of the participants were residents of Addis Ababa. About 37% of the participants had a diploma and above educational level, and 32.6% were employees of government or private companies (Table 1).

**Table 1:** Socio-demographics characteristics of study participants

Variable**, *N* = 319**	N(%)
**Sex**	
Male	163(51.1)
Female	156(48.9)
**Age in years**	
18–27	40 (12.5)
28–40	74 (23.2)
41–50	47 (14.7)
> 50	158 (49.5)
**Educational status**	
Can’t read and write	40(12.5)
Can read and write without formal education	32(10.0)
Primary	67(21.0)
Secondary	63(19.7)
Diploma and above	117(36.7)
**Employment status and type**	
Government or private company employee	104(32.6)
Merchant	12(3.8)
House wife	56(17.6)
Daily laborer	5(1.6)
Student	46(14.4)
Unemployed	96(30.1)

### Clinical and treatment related characteristics

The majority (63%) of the study participants were treated for type-II DM. More than half (51.4%) of the participants claimed they had lived with DM for more than 10 years (mean: 12.6 ± 8.3 years). Regarding the duration of insulin therapy, more than one third (37.6) were taking insulin for more than 10 years. With regard to medication use, 169 of type-II DM patients are taking metformin. Among the study participants, the majority (62.1%) were on NPH insulin therapy. A significantly higher participants 291(91.2%) in this study took insulin two times per day. Of all the participants, 302(94.7%) have ever experienced hypoglycemia and only 103 (32.3%) have noticed injection-related complications like rash, swelling, or bleeding after using insulin (Table 2). Another observation in this study was that only 10% of the study participants were dependent on family members for injections, but most of them (82.1%) injected the insulin by themselves. In addition to their blood sugar medications and sugar restriction to manage their blood sugar level, 39.8% and 30.7% of patients practiced physical exercise and salt restriction, respectively.

**Table 2:** Clinical and treatment related characteristics of studied patients

Variable, *N* = 319	N(%)
Type of Diabetes mellitus	
T1DM	118(37.0)
T2DM	201(63.0)
Duration of DM (Years) Mean ± SD	12.6 ± 8.3
≤ 1	10 (3.1)
> 1–5	47 (14.7)
> 5–10	89 (30.7)
> 10	164 (51.4)
Duration of Insulin therapy (Years) Mean ± SD	10.0 ± 8.0
≤ 1	38 (11.9)
> 1–5	73 (22.9)
> 5–10	88 (27.6)
> 10	120 (37.6)
Type of insulin patient currently taking	
Intermediate acting insulin (NPH)	198(62.1)
Both NPH and regular/rapid acting insulin	121(37.9)
Frequency of insulin administration	
Once per day	291(91.2)
Twice per day	28(8.8)
Support for the injection	
Family member	32(10)
Themselves	263(82.1)
Both	24(7.5)
Non-pharmacological interventions practiced in addition to sugar restriction	
Exercise	127(39.8)
Avoid fatty meals	37(11.6)
Salt restrictions	98(30.7)
Nothing	57(17.9)
Patients taking metformin	169(53.0%)
History of hypoglycemia	302(94.7)
Injection-related complications like rash, swelling, or bleeding after using insulin	103(32.3)

*T1DM* type-I diabetes mellitus, *T2DM* type-II diabetes mellitus

### Insulin injection practice

The common injection site amongst the studied patients was abdomen alone (88, 25.1%) followed by arm alone (76, 23.8%) and both at thigh and arm (52, 16.3%). There were roughly equal numbers of patients who did (159) and did not (160) leave the needle in their body after injecting insulin. Among those who leave the needle in their body, 47.3% of them leave it for five to ten seconds and the rest more than 10 s. Regarding other components of the correct technical insulin injection practice, 96.9% and 53.9% of patients correctly reported inserting the needle perpendicularly (90 degree) and raising the skin at the site of injection, respectively. More than three fourth of participants (*n* = 247) re-use one needle more than five times (Table 3).

**Table 3:** Technical insulin injection practices of the studied patients

Questions		N(%)
What is the most common area that you inject?	Abdomen alone	80 (25.1%)
	Thigh alone	41 (12.9%)
	Arm alone	76 (23.8%)
	Abdomen & thigh	33 (10.3%)
	Abdomen &arm	37 (11.6%)
	Thigh & arm	52 (16.3%)
Do you leave the needle in your body after injecting insulin?	Yes	159 (49.8)
	No	160 (50.2)
How long do you keep it?	Five to ten seconds	151 (47.3)
	Ten to 60 s	8 (2.5)
How many times do you reuse the needle?	One to five times	71 (22.3)
	More than five times	247 (77.4)
Do you make skin fold while injecting?	Yes	172 (53.9)
	No	147 (46.1)
What is the angle that you use to inject insulin?	Inclined (45 degrees)	3 (0.9)
	Perpendicular (90 degrees)	309 (96.9)
	Perpendicular (90 degrees), Inclined (45 degrees)	7 (2.2)

### Insulin handling and injection experiences and practices of participants

**Fig. 1:**
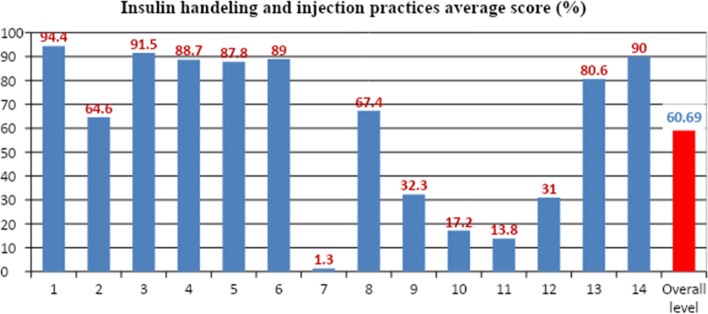
Insulin handling and injection experiences and practices

The median insulin storage and injection practice level of study subjects was 38 out of 56. Most of the participants 234(73.4%) had fair insulin storage and injection practice. The majority of the patients (91.5%) mixed the cloudy insulin NPH prior to use. About 17.2% of patients had ever injected their insulin through their clothes (Fig. 1).

### Association between insulin injection practice score and patient characteristics

For the different educational levels, patients who completed primary and secondary education (Median = 37) had better practice levels than those who can't read and write (Median = 36). Those who achieved a diploma and above (Median = 40) exhibited higher practice levels than those who can read and write without formal education (Median = 37). Respondents aged 18–27 (median score = 40) had better practice than those aged > 50 (median score = 38). The One-way ANOVA conducted using Kruskal Wallis H test showed that there was a statistically significant insulin handling practice score difference between age groups (*p* < 0.0001), educational status (*p* < 0.0001), occupation and years since diagnosis made (*p* = 0.011) (Table 4).

**Table 4:** One way ANOVA using Kruskal Wallis H test to assess correlation between insulin injection practices score and predictor variables among respondents on follow up at TASH

Variable	Practice score				
	**Median (IQR)**	**Mean**	**Test Statistics (X2), (df)**		***P*-value**
Age					
18–27	40(3)	38.97			
28–40	37(5)	36.35			
41–50	40(6)	39.04			
> 50	38(4)	36.32	23.78(3)		< 0.0001
Educational status					
Can’t read and write	36(3)	35.35			
Can read and write without formal education	37(4)	37.95			
Primary	37(6)	33.95			
Secondary	37(6)	36.85			
Diploma and above	40(5)	39.49	53.4(4)		< 0.0001
Occupation					
Government or private company employee	39(4)	38.13			
Merchant	37(14)	32.83			
House wife	36.5(5)	35.1			
Daily laborer	33(3.5)	34.4			
Student	38(3)	38.3	24.24(5)		< 0.0001
Unemployed	38(4.75)	37.12			
Years of insulin Therapy					
≤ 1	37.5(12)	33.81			
> 1–5	38(6)	37.4			
> 5–10	38(4)	37.76			
> 10	38(4)	37.42	2.48(3)		0.478
Years of disease					
≤ 1	38(2)	38.6	11.08(3)		0.011
> 1–5	35(12.75)	33.81			
> 5–10	38(5)	37.5			
> 10	38(4)	37.61			
Type of diabetes mellitus					
Type I	38.81(4)	39	< 0.0001		
Type II	35.96(6)	37	15.56(1)		

### Health related quality of life of patients

In terms of all the domains in the EQ-5D-5L questionnaire namely Mobility, Self-care; Usual activities, Pain/discomfort, and Anxiety/depression, the frequency of restrictions is found to increase across the different age groups of respondents. There is almost no restriction observed in all of the domains for those aged 18–26 (Table 5). The utility scores of the study participants ranged from -0.04 to 1 with mean (SD) utility score of 0.89(± 0.19).

**Table 5:** HRQOL using EQ-5D-5L dimensions according to age category

Variable	All	Age			
		18– 27	28–40	41- 50	> 50
**Mobility**					
No problems	238(74.6%)	32(88.9%)	70(94.6%)	33(70.2%)	103(65.6%)
Slight problems	43 (13.5%)	4(11.1%)	0(0.0%)	11(23.4%)	28(17.8%)
Moderate problems	20 (6.3%)	0(0.0%)	4(5.4%)	0(0.0%)	16(10.2%)
Severe problems	13 (4.1%)	0(0.0%)	0(0.0%)	3(6.4%)	10(6.4%)
Incapacity	0 (0.0%)	0(0.0%)	0(0.0%)	0(0.0%)	0(0.0%)
**Self-care**					
No problems	262 (82.1%)	36(100%)	70(94.6%)	34(72.3%)	122(77.2%)
Slight problems	28 (8.8%)	0(0.0%)	0(0.0%)	6(12.8%)	22(13.9%)
Moderate problems	20 (6.3%)	0(0.0%)	4(5.4%)	7(14.9%)	9(5.7%)
Severe problems	5 (1.6%)	0(0.0%)	0(0.0%)	0(0.0%)	5(3.2%)
Incapacity	0(0.0%)	0(0.0%)	0(0.0%)	0(0.0%)	0(0.0%)
**Usual activities**					
No problems	230 (72.1%)	36(100%)	66(89.2%)	33(70.2%)	95(60.1%)
Slight problems	25 (7.8%)	0(0.0%)	4(5.4%)	4(8.5%)	17(10.8%)
Moderate problems	40 (12.5%)	0(0.0%)	4(5.4%)	4(8.5%)	32(20.3%)
Severe problems	7 (2.2%)	0(0.0%)	0(0.0%)	6(12.8%)	1(0.6%)
Incapacity	13 (4.1%)	0(0.0%)	0(0.0%)	0(0.0%)	13(8.2%)
**Pain/discomfort**					
No	175 (54.9%)	31(86.1%)	56(75.7%)	23(48.9%)	65(41.1%)
Slight	85 (26.6%)	5(13.9%)	14(18.9%)	7(14.9%)	59(37.3%)
Moderate	31 (9.7%)	0(0.0%)	0(0.0%)	10(21.3%)	21(13.3%)
Severe	24 (7.5%)	0(0.0%)	4(5.4%)	7(14.9%)	13(8.2%)
Extreme	0(0.0%)	0(0.0%)	0(0.0%)	0(0.0%)	0(0.0%)
**Anxiety/depression**					
No	223 (69.9%)	36(100%)	57(81.4%)	21(44.7%)	109(70.8%)
Slight	63 (19.7%)	0(0.0%)	9(12.9%)	22(46.5%)	32(20.8%)
Moderate	21 (6.6%)	0(0.0%)	4(5.7%)	4(8.5%)	13(8.4%)
Severe	0(0.0%)	0(0.0%)	0(0.0%)	0(0.0%)	0(0.0%)
Extreme	0(0.0%)	0(0.0%)	0(0.0%)	0(0.0%)	0(0.0%)

### Correlation between EQ VAS score and years of insulin use and duration of DM

**Fig. 2:**
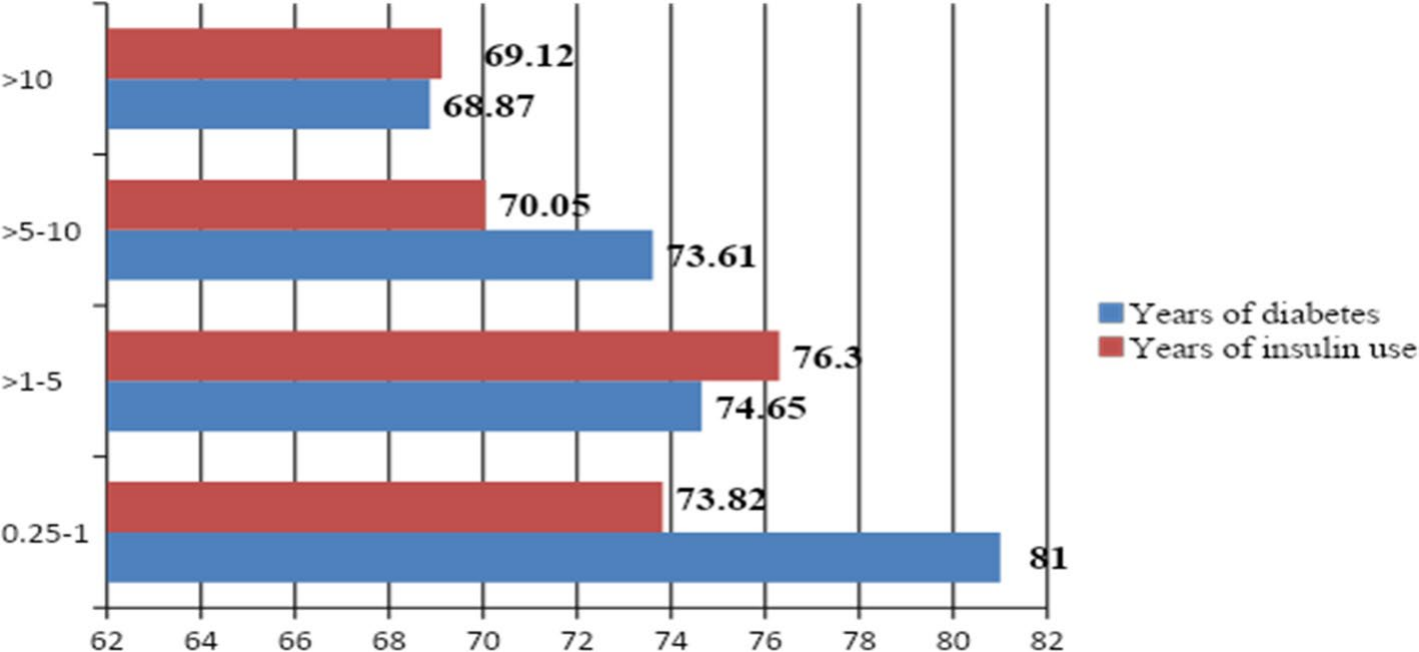
EQ VAS Average score classified based on both diabetes duration and insulin use duration

The average values of EQ VAS score in this study population is seen to be the lowest (68.87) in those patients who have lived with diabetes for more than 10 years and becomes higher as the years of diagnosis decreases. A lower EQ VAS value was also observed in patients who lived more years with diabetes versus those who lived relatively fewer years (Fig. 2).

**Fig. 3:**
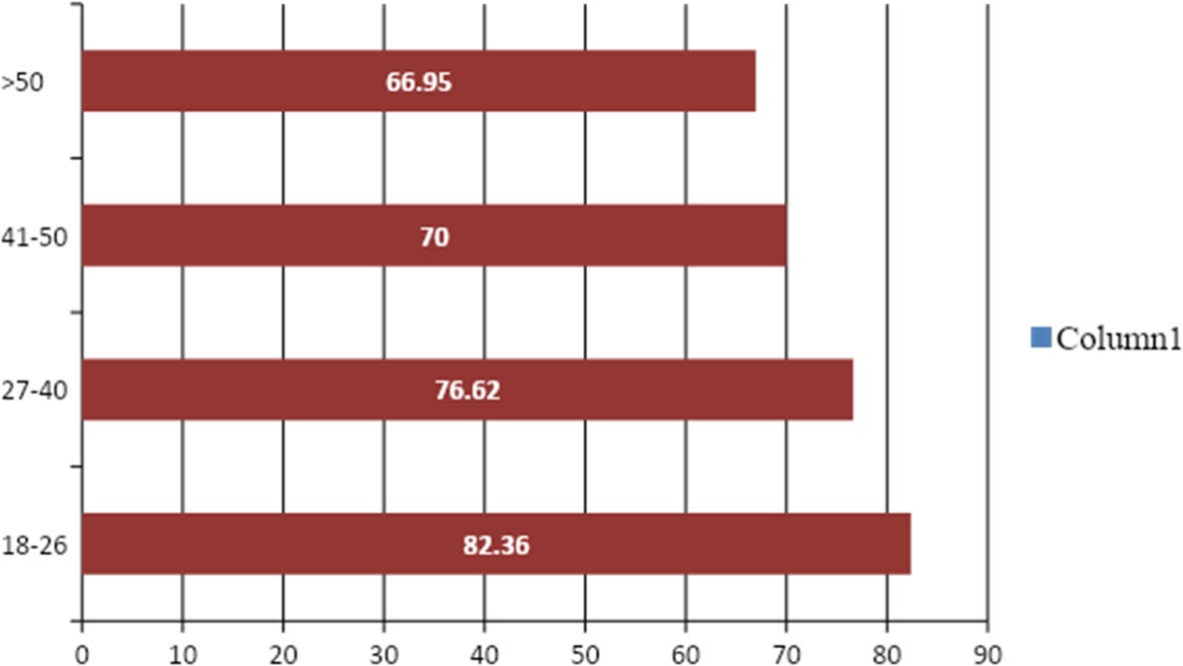
EQ VAS Average score classified based on age

The average EQ VAS score appears to rise as respondents' ages decrease. Subjective health assessment (EQ VAS) was significantly lower in respondents within the age groups > 50 compared to the younger age groups (*p*-value < 0.0001) (Fig. 3).

### Factors affecting health-related quality of life

A total of six variables were included in the bi-variate analysis to investigate factors associated with EQ VAS score of HRQOL. Out of the six variables, only the four variables (two continuous variables namely age and duration of diagnosis in years; and two categorical variables namely sex and type of DM) showed a statistically significant association with HRQOL of patients who are taking insulin therapy. These variables were utilized in multivariate linear regression analysis, and only two variables i.e. sex and type of DM significantly affected HRQOL of patient treated with DM through Enter methods of multivariate linear regression method (Table 6).

**Table 6:** predictors of health-related quality of life

Variable	Crude β-coefficient (95% CI)	*p*-value	Adjusted β-coefficient (95% CI)	*p*-value
Age in years	-0.299(-0.404, -0.194)	< 0.0001	-0.085(-0.254, 0.083)	0.321
Sex	-6.08(-9.23, -2.92)	0.002	-5.423(-8.63, -2.21)	0.001
Duration since insulin therapy	-0.187(-0.388, 0.013)	0.067	-0.132(-0.705, 0.441)	0.650
Duration since diagnosis of diabetes mellitus	-0.306(-0.498, -0.114)	< 0.0001	-0.06(-0.65, 0.529)	0.841
Type of diabetes mellitus	11.69(8.62, 14.78)	< 0.0001	9.04(4.23, 13.85)	< 0.0001
Insulin handling practice score out of 56	0.386(0.119, 0.653)	0.005	0.123(-0.148, 0.393)	0.372

All standardized residuals in the models were normally distributed (*p* < 0.05), meeting the assumptions of the linear regression model. The multiple linear equation became; predicted overall HRQOL = 72.37 + (-5.42 (female)) + (-0.085 (age in years)) + 9.04 (type-I DM) + (-0.06 (duration since diagnosis of DM)) + (-0.132 (Duration of insulin treatment)) + 0.123 (practice score). Making other predictor variables in the equation constant, the interpretation for the result in the equation is as follows. The value indicated that as the number of type-I DM patients increases by one, HRQOL of patients’ increases by 9.04 units. In addition, as the number of female patients increases by one, the HRQOL of patients decreases by 5.423 units (Table 6).

## Discussion

Diabetes mellitus is a global health issue and the compliance of patients with their medical treatment is crucial to reduce the occurrence of complications. Insulin can be used as monotherapy or in combination with other therapies to control blood glucose levels. However, the maximal benefit of insulin depends on its appropriate administration or injection [17].

Reusing the insulin syringe needle is among the most serious insulin injection-related problems that affect glycemic control [18]. In this study, it was noted that all of the respondents reused the needle more than once, with the fact that the majority (77.4%) used a single needle five to ten times which is similar with study conducted in Iran [22]. This finding is much higher than Algerian patients, who appear with 52% over-reuse of the needle [19]. One of the major reasons for the reuse of the needle is the scarcity of insulin syringes in health facilities. Due to shortage of the financial means to purchase and utilize the needle as needed, patients are forced to use one syringe repeatedly until it becomes dull or deformed. This is bad practice as it increases the risk of injection-related complications like rash, LH, bleeding, and discomfort [23]. The study showed that about one third of the patients noticed injection-related complications like rash, swelling, or bleeding after injecting insulin, which is less than the Bangladesh patients, where injection-related complications were reported by almost half of the patients [12]. This variation in the magnitude of noticed injection related complication might be due to difference sample size, study site and socio-demographics characteristics of the participants.

The abdominal region is the preferred site for insulin injections around the world [19]. Similarly, in the current study about 25.1% of the respondents claiming they prefer this site. In contradicting to current study, the arms and thighs were more frequently used as a single injection site by Bangladesh [12] and India [24] patients. Even though LH is considered to be more common in the abdominal area than elsewhere, the lower risk of intramuscular injection and more rapid absorption due to the presence of a thick subcutaneous fat layer have made this site the first choice for insulin injection [25, 26].

In this study almost half of the respondents (49.8%**)** leave the needle in their body after injecting insulin, while only 39% of the Canadian patients reported leaving the pen needle in their skin for 10 s [27] and only a quarter of Algerian respondents respected the recommendation [19]. Although it is better compared to the others, it is not enough, especially considering patients administering higher insulin doses. Mainly to increase the probability that the full insulin dose is delivered and to prevent medication leakage, the patient should leave it for at least 10 s before withdrawing the needle [28].

A skin lift may be formed prior to injection, especially by those using bigger size syringes to reduce the risk of intramuscular injection, which increases the variability of insulin absorption and may impair glycemic control [29]. In this cross-sectional study, 53.9% of the respondents claimed they form skin folding while injecting the needle, which is not much different from Saudi Arabian patients (53.1%) [17]. This again needs to be taken into account, and patients need to be informed about this practice by health care professionals since it could be one of the contributing factors for poor glycemic control among patients.

Regarding insulin handling and injection practice, 12.9% of the respondents in this study had good practice. While only 1.2% of patients in the North West primary hospitals of Ethiopia perform good practice [14]. This low level of good practice is associated with low level of awareness, lack of jobs, elder age, type of disease and long disease duration. A study conducted in French backs the current findings, which reveal that younger age, male gender, higher income, and higher educational level are related with improved quality of life [15]. In fact, each patient must perform appropriate storage and handling of the insulin for a better outcome. Even though most of the respondents say that they mix the cloudy insulin NPH well prior to use, only a few of them demonstrated the proper way of mixing it by rolling, while others simply shake the cartridge like any other medicine container. This is one of the bad habits that may result in inconsistency in the preparation and later compromise insulin efficacy.

This study also showed that most of the respondents (89%) inspect the insulin before injection. Similarly, more than three-fourth of study participants in Iran didn’t give attention even for the expire date [22]. This is necessary because as time goes by, some insulin defect indicator (like, floating of particles in NPH or color changes in regular insulin) might be noticed, which indicates the loss in potency [30]. This is true, particularly for those who use both NPH and regular insulin. Their major reason was during mixing some use only a small dose of regular insulin. More than half of the respondents have said they use the opened insulin vial after 28 days. It is not generally a good practice to store insulin after it has been opened for 28 days because insulin become less effective 28 days after being opened, even though the unopened insulin can be kept until the expiry date [31].

The use of alcohol to clean the top of the vial as well as the injection site is amongst the least common practices of our respondents which is similar with Indian study participants [24], and Nepal [18]. Some of the common reasons were affordability issues, being told by health professionals that they don't need to use alcohol, and not at all being informed about injection site cleaning using alcohol. Even though it's not mandatory, skin cleaning is usually recommended before an insulin injection to prevent infection of the injection site [32].

Injection site rotation is important in order to avoid a build-up of fatty tissue, which can occur when shots are always given in the same place. The build-up of fatty tissue can change how quickly insulin is absorbed from the skin, which may, in turn, affect blood glucose levels [33, 34]. In the current study, 90% of patients rotate the injection sites, while only 55.1% of Saudi Arabian patients perform rotation [17]. The high magnitude of rotation of injection site practice among current study participants might be over exaggerated due to cross sectional study design, which is the claim of study participants. It might not be the correct rotation.

The self-reported outcome of the EQ-5D-5L with EQ-VAS questionnaire was used in this study to evaluate the quality of life of diabetes patients. From this, we have seen that regardless of most of the patients having a fair practice of insulin use and also taking other measures like exercise and salt restriction in their diet to manage their blood glucose level, their quality of life assessed with the EQ 5D-5L scores shows a decline with the increase in age. Similarly the study conducted in Poland also showed that the lower age was associated with better quality of life while patients of older age have more severe restrictions [35]. In this study as the years of insulin use and duration of DM increase the EQ VAS score was also become lower which is similar with other study [36–38].

From the linear regression of multiple variables with the EQ-VAS score, it was seen that type II diabetes patients have lower HRQOL compared to type I diabetes patients. This might be due to patients negative appraisals of insulin therapy such as fear of injecting, worries about gaining weight, worry about the impact of insulin therapy on the social environment when shifted from oral [39]. This could mainly be related to their taking additional drugs (metformin) and/or others, which may affect compliance with drug therapy. On the other hand in the study conducted in Uganda showed that no significant difference were seen among the patients [40]. In addition in current study female gender was associated with lower HRQOL which similar to study conducted in French and Poland [15, 35].

### Limitations

Even though this study brings important information about insulin injection practice and HRQOL among adult diabetes patients, it has some limitations. This study was conducted only on a conveniently selected number of patients, which makes it difficult to generalize about both the practice and quality of life of all the diabetes patients treated with insulin. The small sample size might affect the power of the statistical test. Due to the cross-sectional nature of the study, no temporal association can be drawn from this study. Due to the quantitative nature of the study; in-depth understanding and verification of the problem is impossible.

## Conclusion

In the present study at the TASH diabetes clinic, it was determined that diabetes patients generally had fair practices in insulin handling, storage, and administration techniques. It was observed that the practice of insulin injections was significantly influenced by patient characteristics such age, education level, occupation, the type of diabetes, and the duration of the disease. There was a statistically significant relationship between the HRQOL with sex and the type of diabetes mellitus.

## Data Availability

All data generated or analyzed during this study are included in this published article [and its supplementary information files].

## Abbreviations

AD: Anxiety/depression
DM: Diabetes Mellitus
EQ VAS: European quality of life visual analog scale
EQ-5D-5L: European quality of life 5 dimensions 5-level version
HRQOL: Health Related Quality of Life
LH: Lipohypertrophy
MO: Mobility
PD: Pain/discomfort
SC: Self-care
TASH: Tikur Anbessa Specialized Hospital
Type I-DM: Type I diabetes mellitus
Type II-DM: Type II diabetes mellitus
UA: Usual activities
